# A comprehensive approach to community recruitment for clinical and translational research

**DOI:** 10.1017/cts.2018.324

**Published:** 2018-11-27

**Authors:** Andrew L. Sussman, Carla Cordova, Mark R. Burge

**Affiliations:** 1 Department of Family & Community Medicine, University of New Mexico Health Sciences Center, Albuquerque, NM, USA; 2 Clinical and Translational Science Center, University of New Mexico Health Sciences Center, Albuquerque, NM, USA; 3 Department of Internal Medicine/Endocrinology, University of New Mexico Health Sciences Center, Albuquerque, NM, USA

**Keywords:** Clinical trials, clinical and translational research, recruitment

## Abstract

Recruitment and engagement for clinical and translational research is challenging, especially among medically underserved and ethnic or racial minority populations. We present a comprehensive model developed through the Clinical and Translational Science Center at the University of New Mexico (UNM) Health Sciences Center that addresses 3 critical aspects of participant recruitment. The components of the model are: (1) Recruitment from within UNM to UNM-centered studies, (2) recruitment from within UNM to community-based studies, and (3) recruitment from outside UNM to UNM-centered studies. This model has increased research participant recruitment, especially among medically underserved populations, and offers generalizable translational solutions to common clinical and translational research challenges, especially in settings with similar demographic and geographic characteristics.

## Introduction

One of the main reasons that clinical and translational research (CTR) is so challenging is because efforts to recruit participants often fail [1, 2]. A report from the Institute of Medicine has identified factors that contribute to the failure of adequate participant recruitment to clinical trials: (a) Lack of awareness among physicians and patients that relevant trials are available; (b) lack of awareness of the benefits of engaging in clinical trials; (c) maintaining clinical equipoise, especially when treatment arms are very different and patients or physicians have strong preferences for one therapy over another; and (d) maintaining equipoise for pragmatic trials when the treatment evaluated is widely available and covered by outside payers [3].

Such challenges may be exacerbated among medically underserved and ethnic or racial minority populations given concerns about mistrust of research and the difficulties these groups face regarding clinical trial enrollment and retention [4, 5]. To address these barriers, the Clinical and Translational Science Center (CTSC) at the University of New Mexico Health Sciences Center (UNM HSC) has developed a multipronged, comprehensive approach to address 3 critical aspects of participant recruitment:

1. Recruitment from within UNM to UNM-centered studies: The participant recruitment service (PRS).
2. Recruitment from within UNM to community-based studies: The community engagement and research core (CERC).
3. Recruitment from outside UNM to UNM-centered studies: The community health specialist (CHS).

The purpose of this report is to describe each component of this innovative model in an attempt to generalize strategies toward enhancing clinical and translational research recruitment for widespread application.

## A 3-Pronged Approach

### Recruitment from Within UNM to UNM-Centered Studies: The PRS

UNM CTSC has devised a novel method to screen the electronic health record (EHR) of the UNM hospitals and clinics to identify patients who may qualify for clinical studies of all types, and to contact those patients in order to gauge their interest. The identification of such individuals is complicated by existing Health Insurance Portability and Accountability Act (HIPAA) rules and a need to protect patient privacy. Although the use of EHRs has become commonplace in clinical research, the PRS is innovative because it provides a mechanism to “cold-call” prospective participants in a manner that is respectful of their rights, compliant with relevant national, state and local policies and laws, and does not require the involvement of the participant’s primary care provider [6–9]. Specifically, the PRS employs an “honest contactor” method that allows trained CTSC personnel to contact prospective participants identified from an EHR search. These Honest Contactor agents represent the institution and are independent of the investigators and the protocols that they are calling about, and as such, this work has been designated by the UNM HSC institutional review board (IRB) and the privacy officer as “work preparatory to research,” and is therefore allowable under HIPAA.

**Fig. 1:**
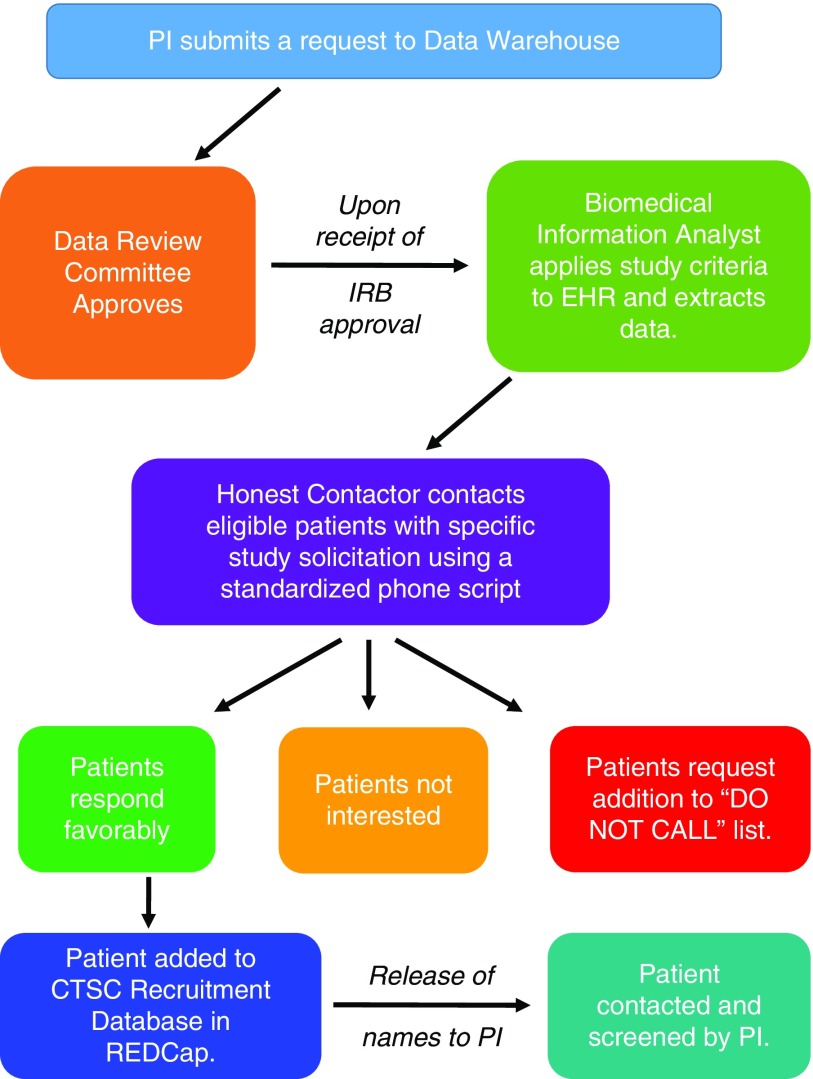
Schematic representation of the Participant Recruitment Service at the University of New Mexico Clinical and Translational Science Center (CTSC). EHR, electronic health records; IRB, Institutional Review Board; PI, Principal Investigator; REDCap, real-time electronic data capture tool.

During an unsolicited telephone call, the honest contactors briefly describe the study in question to assess a patient’s interest in participating. Contact information is forwarded to study-specific personnel for all patients who express positive interest. This methodology was developed with institutional leadership. The notice of privacy practices from the UNM Health System states that, “We can use or share your information for health research” (version date May 3, 2017). The use of protected health information (PHI) that resides in the UNM EHR for the PRS has been vetted and approved by the chief medical information officer, the HIPAA privacy officer, the executive chair of the human research review committee (i.e., the IRB), and the CTSC regulatory affairs manager as being consistent with this policy. Fig. 1 shows the workflow process for the PRS.

In order to make use of the PRS as user-friendly as possible, the investigator makes data search requests using an online form detailing information about inclusion and exclusion criteria and the EHR data to be extracted. A check box on the IRB application indicates plans to use the PRS as a method of recruitment, and no calls are made until IRB approval is received. To ensure that the language of the telephone script is appropriate, the patient literacy division at UNM hospital reviews a standardized script specific to each study during IRB review, and the script is IRB approved. The initial contact provides general study information and seeks patient permission to be contacted by study-specific personnel. The PHI of interested patients is stored in a secure, password-protected database in real-time electronic data capture tool (i.e., REDCap), and links to this REDCap list are provided to study-specific personnel. Names and contact information for interested patients are released in REDCap to such personnel for a period of 5 days, and the list may not be downloaded or saved for future trials.

If a patient desires not to be contacted again, his or her information is added to a standing “Do Not Call List” maintained by the regulatory affair manager. This critical step enables the UNM CTSC to serve as the responsible party and gatekeeper for all PRS calls. Approximately 1.5% of individuals contacted elect to be added to the Do Not Call List. As shown in Table 1, recruitment for UNM CTSC clinical studies that use PRS is increasing, but only a small number of investigators are currently using the service. Our observed enrollment rates of 3–8% of individuals who respond affirmatively to the initial PRS call is superior to the rates of enrollment that typically occur with mass mailing strategies [10–12]. Total study accrual adequately represents the population of New Mexico (NM), with 48% being Hispanic participants, 24% under the age of 18, and 25% above the age of 60.

**Table 1:** Results of a comprehensive, 3-pronged approach to recruitment for clinical and translational research studies at the University of New Mexico Clinical and Translational Science Center over the last 3 grant years

	Grant year (calendar year)		
	6 (2015)	7 (2016)	8 (2017)
Participant recruitment service			
Number of protocols using the service	2	5	4
Number of calls made	1663	1542	7887
Number of contacts made (% of those called)	539 (32%)	374 (24%)	834 (11%)
Number of patients responding affirmatively (% of those contacted)	398 (74%)	235 (63%)	672 (81%)
Number of enrolled (% of those responding affirmatively)	31 (8%)	7 (3%)	41 (6%)
Number of “Do Not Call” requests (cumulative)	73	91	170
Community engagement and research core			
Number of protocols using the service	22	22	24
Number of enrolled	272	642	294
Number of geriatric participants enrolled (>64 years) (% of total)	24 (9%)	91 (14%)	62 (21%)
Number of Hispanic/Latino participants enrolled (% of total)	47 (17%)	185 (29%)	144 (49%)
Number of Native American participants enrolled (% of total)	18 (7%)	39 (6%)	35 (12%)
Number of rural participants enrolled (% of total)	3 (1%)	38 (6%)	126 (43%)
Average number of months to full enrollment	19	12	16
Community health specialist			
Number of protocols using the service	0	2	4
Number of community contacts	0	21	33
Number of community health workers registered with the UNM CTSC community health network	0	2	5
Number of partnering clinics	0	5	7

CTSC, Clinical and Translational Science Center; UNM, University of New Mexico.

### Recruitment from Within UNM to Community-Based Studies: The CERC

Engaging communities across NM into all aspects of CTR is a key component of successful participant recruitment. As such, the UNM CTSC formed a CERC to (a) maintain trained research staff with expertise in the full spectrum of research to support expansion of research with communities throughout NM and (b) serve as a conduit for bidirectional communication between communities and researchers. As an example of this concept, the CERC established a process that uses input from local Community Health Councils to identify the healthcare needs and priorities of 33 counties in NM, which are published annually as “Annual County Health Report Cards” [13]. These community health priorities provide critical input for investigators doing CTR and help develop stronger ties between each of these counties and the UNM CTSC. This bidirectional approach ensures active and substantive community stakeholder participation and decision-making about research. This model has the added benefit of enhancing successful enrollment and retention of participants in CTR, particularly in diverse and rural NM communities.

Recognizing the importance of this approach, the UNM CTSC CERC has expanded their community stakeholder participation through development of the integration of special populations (ISP) team. The ISP team is composed of (a) UNM faculty who have experience conducting disease-focused, rural, community-based, ethnically or racially, and/or age-specific studies and (b) community stakeholders, including those from rural, youth and aging-groups, African American, Hispanic, and Native American groups. These stakeholders serve as liaisons to multiple groups related to their respective areas of expertise. The ISP team has 12–15 committee members (50% academic and 50% community stakeholders) who meet once a month to (a) enhance inclusion by reviewing CTR protocols before IRB submission and make recommendations to better integrate special populations in their protocols, as appropriate; (b) identify best practices at other institutions for the integration of special populations that might be deployed at UNM; (c) integrate ISP activities with other community-focused activities to leverage and enhance engagement, communication, education, and involvement with potential research participants and stakeholders; (d) convene stakeholder focus groups to support continuous improvement in integrating special populations; and (e) provide cultural competency training to faculty, staff, and trainees involved in research activities.

Collectively, these CERC-fostered efforts have increased research participation from traditionally underserved populations. Specifically, the percentage of cumulative participant enrollment for CERC studies has steadily increased among geriatric participants (aged >64 years) over the last 3 years of the CTSC’s current grant, as shown in Table 1. In addition, enrollment for CERC studies increased from 272 participants in grant year 6 to 294 participants in grant year 8. Lastly, the average amount of time to reach full enrollment among CERC studies decreased from 18.5 months in grant year 6 to 15.9 months in grant year 8.

### Recruitment from Outside UNM to UNM-Centered Studies: The CHS

The third component of our comprehensive approach to improving participant accrual is to increase participation in clinical studies by individuals not yet engaged with the UNM CTSC or the UNM Health System. In NM, such participants are often rural-dwelling and geographically removed from Albuquerque. In a venture similar to the University of Florida’s HealthStreet program, a new CHS works within the UNM CTSC to interact with communities and link medically underserved individuals to medical and social services, as well as to opportunities to participate in research [14]. The CHS works with the well-established Health Extension Rural Offices program, and similar to the Health Extension Rural Offices program, the CHS networks with community residents at clinics, churches, schools, health fairs, parks, and other public sites and events to connect community members with resources and services that may improve their socioeconomic situation [15].

At the same time, the CHS offers community members an opportunity to register with the existing UNM CTSC Clinical Research Volunteer Registry, a web portal that enables volunteers to provide PHI so that they can be matched to a relevant, ongoing clinical study. This registry was established in 2004 and now contains 627 validated volunteers. By employing a full-time CHS to populate this registry with additional individuals who are interested in research, we anticipate the registry will continue to grow.

Lastly, the CHS is integral in the recently developed community health network (CHN), which supports a new way of promoting opportunities for engaging rural communities into research. The CHN will develop long-lasting community-academic partnerships, facilitate respectful community engagement, and ensure the alignment of community priorities with CTSC resources. The CHN recognizes the value of community health workers (CHWs) in community and clinic settings and establishes partnerships with them to help identify potential participants who agree to be contacted to receive additional information about specific research opportunities that meet their health interests. This new network of CHWs will provide an innovative, efficient, and accessible enrollment approach to research and clinical trials to rural populations throughout NM that are otherwise disconnected from UNM. Because CHWs have the knowledge, experience, and trust of their communities, they are well positioned to facilitate CTSC-community interactions. The ultimate goal of this activity is to enhance the innovation, access, and quality of clinical and translational research among diverse and rural populations.

## Summary and Conclusions

**Fig. 2:**
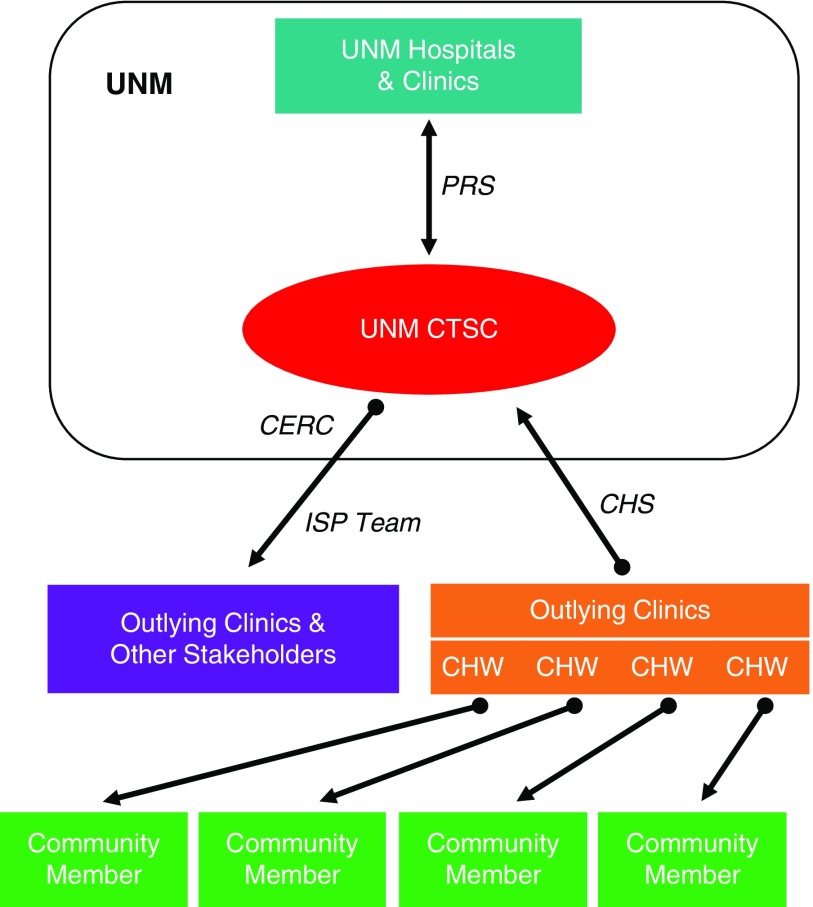
Schematic representation the comprehensive, “3-pronged approach to participant recruitment at the University of New Mexico Clinical and Translational Science Center (UNM CTSC). Although these activities are routinely bidirectional between the research cores and their stakeholders, in the context of this report focusing on study recruitment, we have opted to depict our arrows as unidirectional, when appropriate, for the sake of clarity. CERC, community engagement and research core; CHS, community health specialist; CHW, community health worker; ISP, integrating special populations; PRS, participant recruitment service.

In NM, a mostly rural, minority-majority state, we have developed a multilevel participant recruitment infrastructure through the CTSC, as shown in Fig. 2. These complementary approaches have improved our “in reach” to patients within the UNM system (i.e., the PRS), facilitated CTR by maintaining a staff of trained researchers to assist investigators with community-based recruitment and data collection efforts (CERC), and designated a CHS in diverse community settings to serve as a liaison to connect community members with health resources and to identify participants who are interested in ongoing research opportunities (CHS). This approach combines “low touch,” system-level recruitment strategies with “high touch,” community-based engagement to create mechanisms of bidirectional communication between UNM and underserved communities throughout NM. We have only recently added the CHS component and we will continue to evaluate the effectiveness of this model and identify other opportunities to enhance integration of these components as we expand our research capacity. This model has increased research participant recruitment, especially among medically underserved populations, and offers generalizable translational solutions to common CTR challenges, especially in settings with similar demographic and geographic characteristics.
